# Removal of *p*-Nitrophenol by Adsorption with 2-Phenylimidazole-Modified ZIF-8

**DOI:** 10.3390/molecules28104195

**Published:** 2023-05-19

**Authors:** Yu Zhao, Peiqing Yuan, Xinru Xu, Jingyi Yang

**Affiliations:** 1 International Joint Research Center of Green Energy Chemical Engineering, East China University of Science and Technology, Meilong Road 130, Shanghai 200237, China; 15106122603@163.com (Y.Z.); xrxu86@ecust.edu.cn (X.X.); 2 State Key Laboratory of Chemical Engineering, East China University of Science and Technology, Meilong Road 130, Shanghai 200237, China; pqyuan@ecust.edu.cn

**Keywords:** *p*-nitrophenol, zeolitic imidazolate frameworks, adsorption

## Abstract

Petrochemical wastewater contains *p*-nitrophenol, a highly toxic, bioaccumulative and persistent pollutant that can harm ecosystems and environmental sustainability. In this study, ZIF-8-PhIm was prepared for *p*-nitrophenol removal from petrochemical wastewater using solvent-assisted ligand exchange (SALE) with 2-phenylimidazole(2-PhIm). The ZIF-8-PhIm’s composition and structure were characterised using the XRD, SEM, FT-IR, ^1^H NMR, XPS and BET methods. The adsorption effect of ZIF-8-PhIm on *p*-nitrophenol was investigated with the static adsorption method. Compared to the ZIF-8 materials, ZIF-8-PhIm exhibited stronger π-π interactions, produced a multistage pore structure with larger pore capacity and size, and had increased hydrophilicity and exposure of adsorption sites. Under optimised conditions (dose = 0.4 g/L, T = 298 K, C_0_ = 400 mg/L), ZIF-8-PhIm achieved an adsorption amount of 828.29 mg/g, which had a greater *p*-nitrophenol adsorption capacity compared to the ZIF-8 material. The Langmuir isotherm and pseudo-second-order kinetic models appropriately described the *p*-nitrophenol adsorption of ZIF-8-PhIm. Hydrogen bonding and π-π interactions dominated the *p*-nitrophenol adsorption of ZIF-8-PhIm. It also had relatively good regeneration properties.

## 1. Introduction

Phenolic aromatic compounds are among the most widespread pollutants in the effluents of petroleum and petrochemical industries. These compounds can cause surface water pollution, posing a significant threat to human health and the environment [1,2,3,4]. Of these compounds, *p*-nitrophenol (4-NP) is a nondegradable, bioaccumulative, persistent and highly toxic pollutant. The main methods for *p*-nitrophenol removal from wastewater include adsorption, precipitation, redox reactions, membrane separation and biological methods [5,6,7]. However, adsorption is commonly used because it is safe, efficient and easy to implement [8,9]. Zhao et al. [10] used steel slag to remove *p*-nitrophenol with an adsorption amount of 109.66 mg/g. Dos Santos et al. [11] modified montmorillonite clay, and its *p*-nitrophenol adsorption ability was 122.09 mg/g. According to Alvarez-Torrellas et al. [12], the *p*-nitrophenol adsorption using peach stones was 234 mg/g. There are numerous natural adsorbent sources, but their adsorption capacity is low, making research on novel adsorbents crucial.

Metal–organic backbones (MOFs) are crystalline materials composed of organic linkers and metal ions with high porosity, abundant unsaturated coordination, high chemical stability and large specific surface area [13,14,15]. Lin et al. [16] synthesised HKUST-1, which exhibited an impressive *p*-nitrophenol adsorption capacity of 371 mg/g. Similarly, Miao et al. [17] produced a silver(I)3,5-diphenyltriazole MOF, AgTz-1, which adsorbed 143.5 mg/g *p*-nitrophenol. Zhi et al. [18] produced amino-MIL-53(Al) sandwich-structure membranes with a *p*-nitrophenol adsorption capacity of 297.85 mg/g. Although ZIF-8 is a popular MOF research topic, only a few studies have investigated its interaction with *p*-nitrophenol in water.

The solvent-assisted ligand exchange (SALE) method is an indirect approach to synthesising materials that replace or exchange organic linkers in MOFs, thereby improving certain properties [19,20,21]. Kenyotha K. et al. [22] used the SALE method to modify ZIF-8 for CO_2_ adsorption, which resulted in a higher pore volume and size with increased CO_2_ uptake.

Most MOF materials are microporous, which may prevent the entry of larger anions into the framework cavities and affect their adsorption capacity [23]. ZIF-8 was modified with 2-phenylimidazole using the solvent-assisted ligand exchange method. The introduction of 2-phenylimidazole enhanced its π-π interaction with *p*-nitrophenol. The small number of defects produced during the ligand exchange not only created a multilevel pore structure with an increased average pore volume and pore diameter in ZIF-8-PhIm but also exposed more active sites. ZIF-8-PhIm’s composition and structure were characterised using the XRD, SEM, FT-IR, BET, XPS and 1H NMR methods. The *p*-nitrophenol adsorption effects of ZIF-8 and ZIF-8-PhIm were compared using a static adsorption method. The regeneration performance and adsorption mechanism were also studied. 

## 2. Results and Discussion

### 2.1. Characterisation of ZIF-8-PhIm and ZIF-8

**Figure 1:**
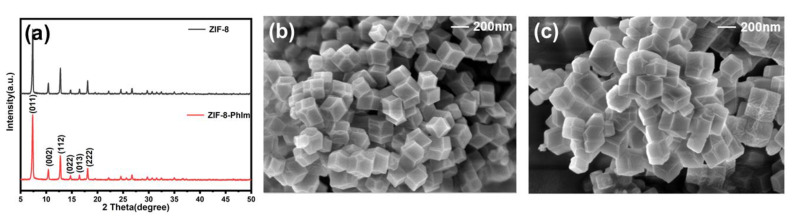
(**a**) XRD results of ZIF-8-PhIm and ZIF-8; SEM images of (**b**) ZIF-8 and (**c**) ZIF-8-PhIm.

Figure 1a illustrates the XRD spectra of the ZIF-8-PhIm and ZIF-8. The XRD spectrum of ZIF-8-PhIm and ZIF-8 were essentially identical at 7.3° (011), 10.4° (002), 12.7° (112), 14.7° (022), 16.4° (013) and 18.0° (222) [24], indicating that the ZIF-8 backbone was preserved. 

Figure 1b,c display the SEM graphics of the ZIF-8-PhIm and ZIF-8. The particle size of the samples increased after the modification, with an average size of approximately 175 nm for ZIF-8 and 229 nm for ZIF-8-PhIm. This was due to the recrystallisation of the ZIF-8 during the exchange process [25].

According to the ^1^H NMR spectral data of ZIF-8-PhIm (Figure S3, Supplementary Materials), 2-phenylimidazole successfully replaced a portion of 2-methylimidazole with an exchange ratio of approximately 1:0.04 [22]. The low exchange rate may be due to the spatial barrier effect of the larger benzene ring, and the exchange occurred mainly on the surface [26].

**Figure 2:**
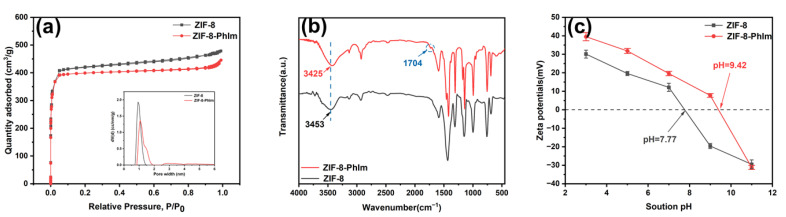
(**a**) N_2_ adsorption and desorption isotherms and pore size distributions of ZIF-8-PhIm and ZIF-8; (**b**) FT-IR results of ZIF-8-PhIm and ZIF-8; (**c**) zeta potential of ZIF-8-PhIm and ZIF-8.

Figure 2a depicts the pore size distributions and N_2_ adsorption–desorption isotherm curves of the ZIF-8-PhIm and ZIF-8. The N_2_ adsorption–desorption isotherm curves conformed to the typical type I isotherm, indicating that the pore structure was mainly microporous [27].

The pore structure parameters and specific surface area are provided in Table 1. The specific surface area of ZIF-8-PhIm (1663 m^2^/g) decreased compared to that of ZIF-8 (1700 m^2^/g), but the pore volume and size (0.85 cm^3^/g; 2.08 nm) of ZIF-8-PhIm increased compared to that of ZIF-8 (0.79 cm^3^/g; 1.85 nm), and the proportion of mesopores increased from 24.05% to 36.47%. This is due to the small number of defects created during the exchange process [28,29,30].

According to the FT-IR image (Figure 2b), the -OH absorption peak exhibited a wave number change from 3453 cm^−1^ to 3425 cm^−1^ in ZIF-8-PhIm, with a noticeable shift towards lower wave numbers and an increase in hydrogen bonding. The characteristic peak at 1704 cm^−1^ belongs to the octave and group frequency bending vibration δCH on the benzene ring, while the weak peak intensity was due to a relatively low exchange ratio of imidazole.

The droplet image (Figure S4, Supplementary Materials) shows that the contact angle of ZIF-8-PhIm (74.42°) decreased by approximately 10° compared to ZIF-8 (84.66°). This reduction was due to the strengthened hydrogen bonding, which promoted the binding of molecules with water molecules, thus enhancing the hydrophilicity. The hydrophilic nature of the ZIF-8-PhIm material facilitated the exposure of the adsorption active sites.

Figure 2c demonstrates that the zeta potential of ZIF-8-PhIm decreased gradually as the pH increased. Its isoelectric point occurred at pH = 9.42, while that of ZIF-8 was at pH = 7.77. At a pH < 9.42 of the solution, the ZIF-8-PhIm surface exhibited a positive charge. The conjugation of the benzene and imidazole rings in 2-PhIm resulted in a decreased electron cloud density on the imidazole ring, leading to the enhanced positivity of Zn.

### 2.2. p-Nitrophenol Adsorption by ZIF-8-PhIm

#### 2.2.1. Effect of Time

**Figure 3:**
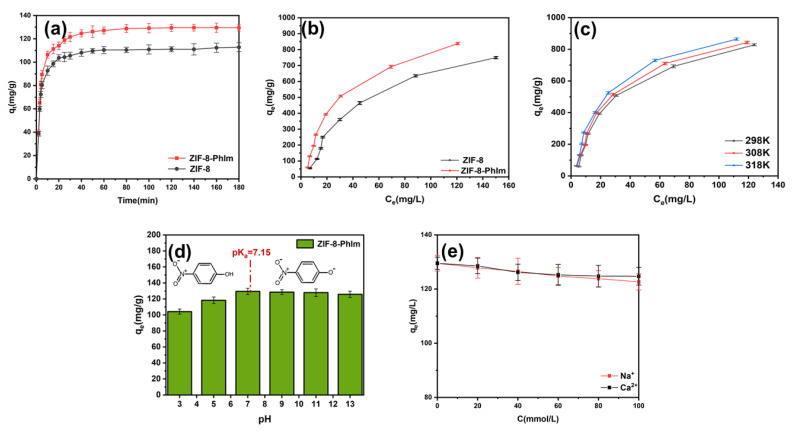
(**a**) Effect of time on *p*-nitrophenol adsorption (T = 298 K; dose = 0.4 g/L; C_0_ = 50 mg/L); (**b**) effect of initial concentration on *p*-nitrophenol adsorption (T = 298 K; t = 180 min; dose = 0.4 g/L); (**c**) effect of temperature on *p*-nitrophenol adsorption (t = 180 min; dose = 0.4 g/L); (**d**) effect of pH on *p*-nitrophenol adsorption (T = 298 K; dose = 0.4 g/L; C_0_ = 50 mg/L; t = 180 min); (**e**) effect of ion concentration on *p*-nitrophenol adsorption (T = 298 K; t = 180 min; C_0_ = 50 mg/L; dose = 0.4 g/L).

As shown in Figure 3a, at T = 298 K and C_0_ = 50 mg/L, the adsorption rate of 4-NP for both adsorbents initially increased rapidly during the first 20 min of contact time and then gradually decreased, reaching an equilibrium after 120 min. This was probably due to the abundance of available adsorption active sites on the adsorbent surface in the early phases. As time progressed, the binding sites became increasingly occupied by *p*-nitrophenol ions, resulting in a slower adsorption rate.

#### 2.2.2. Effect of Initial Concentration 

Figure 3b demonstrates that ZIF-8-PhIm exhibited a greater increase in adsorption with an increasing initial *p*-nitrophenol concentration than did ZIF-8. At a temperature of 298 K, adsorption time of 180 min, and initial concentration of 400 mg/L, the adsorption capacity of ZIF-8-PhIm reached 828.29 ± 6.95 mg/g, which was higher than that of ZIF-8 (749.47 ± 7.29 mg/g) and many previously published adsorbents (Table 2). This was likely due to the multistage pore structure of ZIF-8-PhIm, which features a larger pore capacity and size, increased hydrophilicity, and exposure to more adsorption sites than ZIF-8 materials. 

#### 2.2.3. Effect of Temperature

Figure 3c illustrates that the amount of ZIF-8-PhIm adsorbed increased with temperature in an initial concentration range of 25–400 mg/L. This temperature-dependent variation in the adsorption amount increased with an increasing initial concentration. This was due to the increased thermal motion of the molecules, which facilitated diffusion into the pore size.

#### 2.2.4. Effect of pH

A solution of 0.1 mol/L sodium hydroxide and hydrochloric acid was used to adjust the pH of the *p*-nitrophenol solution. The adsorption capacity of ZIF-8-PhIm at different pH values was determined at T = 298 K and C_0_ = 50 mg/L (Figure 3d). *p*-Nitrophenol had a pKa of 7.15 and existed mainly in the ionic form at pH > 7.15 and molecular form at pH < 7.15. At pH < 9.42, the ZIF-8-PhIm surface was always positively charged. With a pH between 3 and 7, the adsorption capacity was significantly reduced due to the partial damage to the adsorbent structure and loss of active centres [31]. When the pH increased from 7 to 9, *p*-nitrophenol was primarily present in the anionic form, leading to an electrostatic interaction between *p*-nitrophenol and the positively charged surface of ZIF-8-PhIm. However, when the pH was raised from 9 to 13, the negative charge on the ZIF-8-PhIm’s surface gradually increased. Therefore, the *p*-nitrophenol adsorption by ZIF-8-PhIm was favoured by electrostatic repulsion, but the adsorption capacity decreased. Additionally, at higher pH values, excess hydroxyl ions competed with *p*-nitrophenol molecules for adsorption sites, further reducing the adsorption capacity. However, the decrease in the adsorption capacity with an increasing pH from 7 to 13 was minimal, indicating that the electrostatic effect was weak and probably not dominant.

#### 2.2.5. Effect of Ion Concentration

Sodium and calcium ions were selected to examine the selectivity of ZIF-8-PhIm for *p*-nitrophenol adsorption. Figure 3e shows that at a temperature of 298 K and an initial concentration of 50 mg/L, the adsorption amounts decreased from 129.5 mg/g to 122.67 mg/g for sodium ions and 124.72 mg/g for calcium ions when the concentration of coexisting ions was 100 mmol/L. This indicates that certain ions may slightly affect *p*-nitrophenol adsorption, but the effect is minimal.

**Table 2:** Adsorption amount of *p*-nitrophenol on different adsorbents.

Material	Optimised Adsorption Conditions	Adsorption Capacity (mg/g)	Reference
HKUST-1	C_0_ 200 mg/L; T 293 K	371	[16]
MOF-AgTz-1	C_0_ 50 mg/L; T 298 K	143.5	[17]
NH_2_-MIL-53	C_0_ 800 mg/L; T 298 K	297.85	[18]
Platanus leaves	C_0_ 300 mg/L; T 298 K	622.73	[32]
AC-NH_2_-MIL-101(Cr)	C_0_ 200 mg/L; T 298 K	182.3	[33]
PS-CH_2_-[C_2_NH_2_MIm][Br]	C_0_ 10,000 mg/L; T 298 K	1269.8	[34]
ZnAl-layered double hydroxides	C_0_ 120 mg/L; T 298 K	101.6	[35]
MgCo-3D hydrotalcite nanospheres	C_0_ 300 mg/L; T 298 K	625.2	[36]
ZIF-8-PhIm	C_0_ 400 mg/L; T 298 K	828.29 ± 6.95	this work

### 2.3. Isotherms and Thermodynamics

The Langmuir and the Freundlich isotherm models were selected for fitting [37].

(1) $$\mathrm{Langmuir isotherm model}\text{: }\frac{C_{e}}{q_{e}}=\frac{1}{q_{m}K_{L}}+\frac{C_{e}}{q_{m}}$$

(2) $$\mathrm{Freundlich isotherm model}\text{: }q_{e}{=K}_{F}C_{e}^{\frac{1}{n}}$$

where *C_e_*—the *p*-nitrophenol concentration at adsorption balance (mg/L); *q_e_*—the adsorption amount of the ZIF-8-PhIm at equilibrium (mg/g); *K_L_*—the Langmuir adsorption constant (L/mg); *K_F_*—the Freundlich model constant (mg/g) (mg/L)^1/*n*^; and *q_m_*—the theoretical maximum single molecule adsorption amount (mg/g).

**Figure 4:**
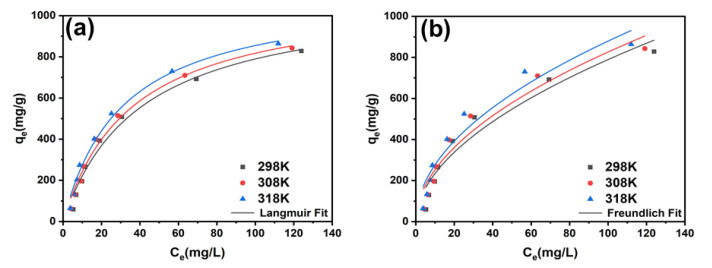
(**a**) Isotherm curves of *p*-nitrophenol adsorption fitting with the Langmuir model; (**b**) isotherm curves of *p*-nitrophenol adsorption fitting with the Freundlich model.

The results of the model fitting, as provided in Figure 4a,b and Table 3, show that the Langmuir isotherm model (R^2^ = 0.981) provided a better description of the adsorption behaviour of ZIF-8-PhIm compared to the Freundlich isotherm model (R^2^ = 0.926). This indicates that the *p*-nitrophenol adsorption by ZIF-8-PhIm was a spontaneous process with a single molecular layer, and the active centres were distributed relatively homogeneously.

Further evaluation of the adsorption process was performed by calculating the thermodynamic parameters.

(3) $$K_{L}=\frac{C_{s}}{C_{e}}$$

(4) $$\Delta G=-{RTlnK}_{L}\;$$

(5) $${lnK}_{L}=\frac{\Delta S}{R}-\frac{\Delta H}{RT}\;$$

where *R*—gas constant, 8.314 J/(mol·K); *T*—temperature (K); *K_c_*—adsorption equilibrium constant; *C_s_*—*p*-nitrophenol concentration adsorbed by the adsorbent at the adsorption balance (mg/L); and *C_e_*—*p*-nitrophenol concentration in solution at the adsorption balance (mg/L).

The thermodynamic calculations shown in Table 4 indicate that the adsorption process was heat-absorbing, as indicated by the positive value of Δ*H* for adsorption (+27.85 kJ/mol). As the value of Δ*H* was less than 29 kJ/mol, this suggests the presence of physical adsorption [38]. Additionally, the negative value of Δ*G* (Δ*G* < 0) indicates that the process of *p*-nitrophenol adsorption was spontaneous. Finally, the positive value of Δ*S* (Δ*S* > 0) suggests that the adsorption process increased entropy.

### 2.4. Adsorption Kinetics

A detailed study of the adsorption kinetics was important to examine the adsorption behaviour of ZIF-8-PhIm on *p*-nitrophenol. Therefore, two kinetic models, the pseudo-first-order model and the pseudo-second-order model, were chosen to fit the experimental data [39,40].

(6) $$\mathrm{Pseudo-first-order equation}\text{: }ln\left(q_{e}-q_{t}\right)=ln\left(q_{e}\right)-k_{1}t$$

(7) $$\mathrm{Pseudo-second-order equation}\text{: }\frac{t}{q_{t}}=\frac{1}{k_{2}q_{e}^{2}}+\frac{t}{q_{e}}$$

where *V*—the solution volume (L); *m*—the adsorbent weight (g); *C_t_*—the *p*-nitrophenol concentration (mg/L) at a certain time (t); *q_t_*—the adsorption amount (mg/g) at a certain time (t); *q_e_*—the amount of adsorption at adsorption balance (mg/g); *k*_1_—the rate constant for the pseudo-first-order model (min^−1^); *k*_2_—the rate constant for the pseudo-second-order model (g/(mg·min)).

**Figure 5:**
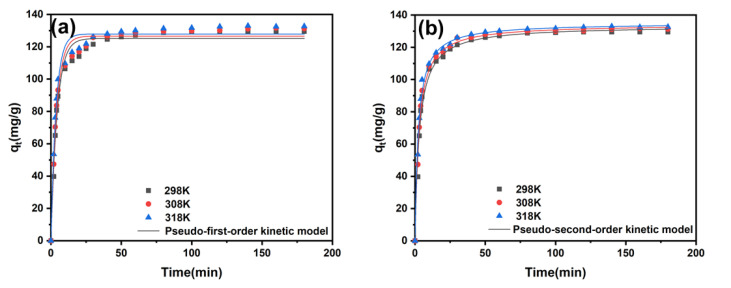
(**a**) Kinetic curves of adsorption fitting with the pseudo-first-order kinetic model; (**b**) kinetic curves of adsorption fitting with pseudo-second-order kinetic model.

The fitting results, provided in Figure 5a,b and Table 5, show that the pseudo-second-order model (R^2^ = 0.989) was better fitted for characterising the adsorption behaviour of ZIF-8-PhIm on *p*-nitrophenol compared to the pseudo-first-order model (R^2^ = 0.969).

Since the pseudo-second-order model was better fitted for *p*-nitrophenol adsorption, the parameters of this model were selected to determine the activation energy. The activation energy was calculated using the Arrhenius equation, which is expressed in Equations (8) and (9):

*k* = *A* × *EXP*(−*Ea*/*RT*) (8)

*In*(*k*) = *ln*(*A*) − *Ea*/*RT* (9)

where *R*—the gas constant, 8.314 J/(mol·K); *k*—the reaction rate constant at a temperature of T (min^−1^); *A*—the finger front factor (min^−1^); *T*—the temperature (K); *Ea*—the reaction activation energy (J/mol).

The activation energy fitting straight line for the adsorption of ZIF-8-PhIm is depicted in Figure S6 (Supplementary Materials). The activation energy obtained from the fitting was 11.54 kJ/mol, which was less than 30 kJ/mol, indicating that the *p*-nitrophenol adsorption process was a typical type of physical adsorption [41].

### 2.5. Adsorption Mechanism

**Figure 6:**
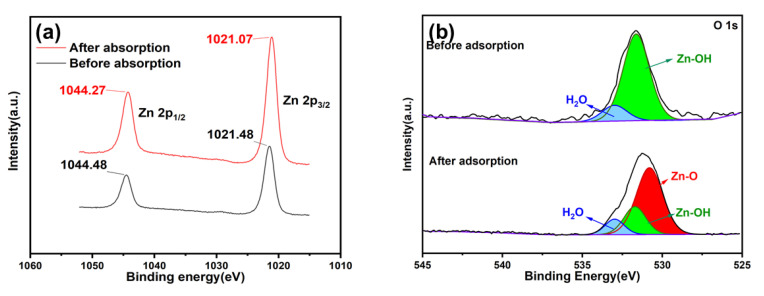
XPS of ZIF-8-PhIm before and after *p*-nitrophenol adsorption: (**a**) Zn 2p; (**b**) O 1s.

The Zn 2p spectrum before adsorption displayed two peaks at 1044.48 eV and 1021.48 eV (Figure 6a), corresponding to Zn 2p_1/2_ and Zn 2p_3/2_ orbitals, respectively. However, after adsorption, these peaks were shifted towards lower binding energies by 0.41 eV and 0.21 eV, respectively. This suggests that the electrons of the *p*-nitrophenol overlapped with the external electrons of Zn^2+^, causing an increase in the density of the outer electrons and enhancing the shielding effect [36].

Figure 6b shows the O 1s spectra. Before adsorption, two peaks were observed at 532.98 eV and 531.71 eV, corresponding to adsorbed H_2_O and Zn-OH, respectively [42]. After adsorption, a new peak was observed at 530.80 eV, corresponding to Zn-O [43]. The reduction in the Zn-OH content and the appearance of the Zn-O peak suggest that *p*-nitrophenol molecules covered Zn-OH and that hydroxyl groups were involved in the ZIF-8-PhIm adsorption on *p*-nitrophenol (Table 6) [44]. The presence of the Zn-O peak was also attributed to the coordination of a partially unsaturated metal (Zn) site in ZIF-8-PhIm with the nitro group in *p*-nitrophenol. Compared to H_2_O, *p*-nitrophenol is more electronegative, providing Zn with numerous electrons in the vacant orbital. Therefore, in an aqueous solution, the partial water molecule coordinated to Zn of ZIF-8-PhIm (Lewis acid) was displaced by *p*-nitrophenol (strong Lewis base), thus forming a coordination bond [36,45,46]. Table 6 shows the binding energies and relative contents in the O 1s profiles of ZIF-8-PhIm before and after *p*-nitrophenol adsorption.

**Figure 7:**
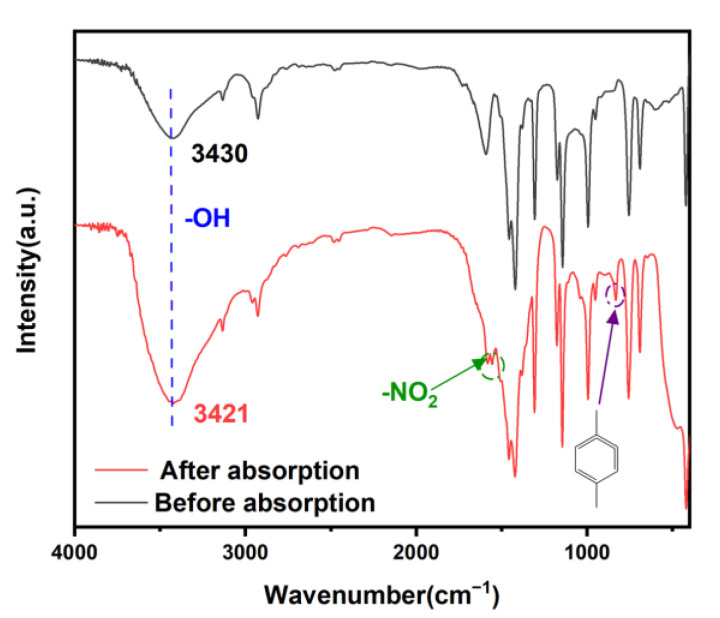
FT-IR spectrum of ZIF-8-PhIm before and after *p*-nitrophenol adsorption.

Figure 7 displays the FT-IR spectra before and after adsorption. The functional groups remained unchanged after adsorption, indicating that the ZIF-8-PhIm structure was unaltered during adsorption. The wave number of 1558 cm^−1^ corresponded to the asymmetric stretching vibration absorption peak of -NO_2_, while the wave number of 833 cm^−1^ corresponded to the para-substitution of the benzene ring, indicating the successful adsorption of *p*-nitrophenol. The -OH absorption peak exhibited variations in the wave number from 3430 cm^−1^ to 3421 cm^−1^ after adsorption, moving towards a lower wave number, indicating hydrogen bond formation. Thus, the hydrogen bonding interaction can be identified as a hydrogen bond formed by Zn-OH with -OH and -NO_2_ [10].

Additionally, the benzene ring’s composition in ZIF-8-PhIm attracts benzene ring-containing compounds through π-π superposition interactions [47,48,49,50].

**Figure 8:**
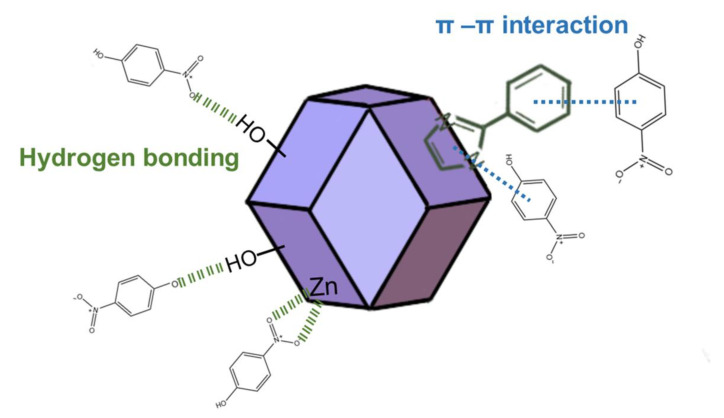
Adsorption mechanism of *p*-nitrophenol on the ZIF-8-PhIm.

Conclusively, the mechanism of *p*-nitrophenol adsorption, as shown in Figure 8, can be attributed to the key interactions, including hydrogen bonding, π-π interaction and weaker electrostatic attraction of the *p*-nitrophenol negative ion to the positive surface of ZIF-8-PhIm. Simultaneously, increasing the pore size and volume of the material increased the hydrophilicity and exposed more active sites for adsorption.

### 2.6. Regeneration Experiment

**Figure 9:**
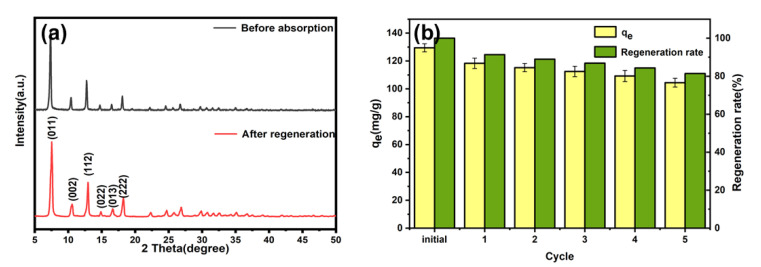
(**a**) XRD of ZIF-8-PhIm before adsorption and after regeneration; (**b**) regeneration efficiency and adsorption capacity of the ZIF-8-PhIm after adsorption–desorption cycles (T = 298 K; dose = 0.4 g/L; t = 180 min; C_0_ = 50 mg/L).

The reusability of the adsorbent is crucial for practical applications, from both an economic and practical perspective. The XRD pattern (Figure 9a) showed that the crystalline shape remained unchanged after regeneration. However, compared to the fresh adsorbent, *p*-nitrophenol adsorption by the regenerated ZIF-8-PhIm decreased from 129.51 ± 2.88 mg/g initially to 104.43 ± 3.13 mg/g after five regeneration treatments (Figure 9b). Therefore, ZIF-8-PhIm demonstrates good reusability.

## 3. Materials and Methods

### 3.1. Chemicals

2-Methylimidazole (2-MeIm), analytical purity, Shanghai Bide Pharmaceutical Technology Co., Ltd., Shanghai, China; zinc nitrate hexahydrate, analytical purity, Shanghai Titan Co., Ltd., Shanghai, China; 2-phenylimidazole (2-PhIm), analytical purity, Shanghai Bide Pharmaceutical Technology Co., Ltd., Shanghai, China; methanol, analytical purity, Shanghai Titan Co., Ltd., Shanghai, China; *p*-nitrophenol, analytical purity, Beijing Bailingway Technology Co., Ltd., Beijing, China; sodium hydroxide, analytical purity, Shanghai Titan Co., Ltd., Shanghai, China; sodium chloride, analytical purity, Shanghai Naisheng Biotechnology Co., Ltd., Shanghai, China; hydrochloric acid, analytical purity, Shanghai Naisheng Biotechnology Co. Ltd., Shanghai, China; calcium chloride, analytical purity, Shanghai Naisheng Biotechnology Co., Ltd., Shanghai, China.

### 3.2. Synthesis of Adsorbent 

ZIF-8 was synthesised using the solvent method. First, 1.49 g of Zn(NO_3_)_2_·6H_2_O (5 mmol) and 1.64 g of 2-MeIm (20 mmol) were dissolved separately in 60 mL of methanol. The two solutions were then mixed and stirred for 24 h at ambient temperature. The product was obtained by centrifuging at 7000 rpm for 30 min and washed thrice with methanol. Finally, the product was oven-dried at 70 °C.

**Figure 10:**
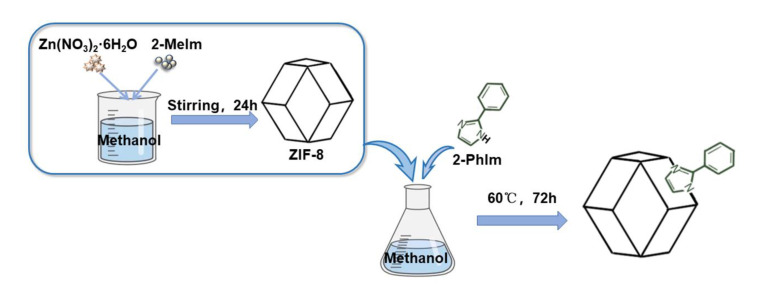
Diagram of the preparation of ZIF-8-PhIm.

For the synthesis of ZIF-8-PhIm, 0.1 g of ZIF-8 was sonicated for 3 min in 20 mL of methanol. The solution was then transferred to a glass vial, and 2-PhIm was dissolved in 30 mL of methanol and added to the ZIF-8 solution. The bottle was sealed and kept for 72 h at 60 °C. After cooling the solution, the formed solid was washed with methanol and separated by centrifugation at 7000 rpm for 30 min. Finally, ZIF-8-PhIm was obtained by drying for 24 h at 70 °C. The molar ratio of ZIF-8 to 2-phenylimidazole was 1:5. The detailed preparation method is displayed in Figure 10.

### 3.3. Characterisation 

X-ray powder diffraction (XRD) measurements were conducted using an X-ray diffractometer (D8 Advance, Bruker Ltd., Karlsruhe, Germany) with a scan rate of 10° per min, a range of 5–90°, and a scan step of 0.01°, using copper alpha radiation of 40 mA and 40 kV. A scanning electron microscope (SEM) (Nova Nano SEM 450, EFI, Hillsboro, OR, USA) with an acceleration rate of 20 kV was used to obtain the SEM images. X-ray photoelectron spectroscopy (XPS) was conducted with an X-ray photoelectron spectroscope (ESCALAB 250 XI, Thermo Fisher Scientific, Waltham, MA, USA) at a 12 kV and 6 mA current under Al Kα radiation. BET testing was performed with a fully automated specific surface and porosity analyser (ASAP 2460, Micromeritics, Atlanta, GA, USA). The FT-IR analysis was performed with an infrared spectrometer (Nicorette 6700, Thermo Fisher Scientific, Waltham, MA, USA). The hydrogen spectroscopy (^1^H NMR) was performed using a 600 M NMR spectrometer (Ascend 600, Bruker Ltd., Karlsruhe, Germany) with deuterated trifluoroacetic acid as the deuterium reagent. The contact angles (CAs) were measured using an optical contact angle meter (OCA 20, Dataphysics, Stuttgart, Germany) to determine the contact angle of a sample with water. The zeta potential was measured with a Zetasizer analyser (Microtrac S3500SI, Magik Instruments Co., Orlando, FL, USA).

### 3.4. Static Adsorption

In the adsorption experiments, 0.4 g/L of adsorbents was added to 25–400 mg/L of 4-NP solution and adsorbed for 180 min. The experimental procedure involved weighing precisely 0.01 g of adsorbent (ZIF-8 or ZIF-8-PhIm), adding it to a 50 mL conical flask. Then, 30 mL of *p*-nitrophenol solution, with varying initial concentrations, was added to the flask. The flask was then capped with a rubber stopper and transferred quickly to a thermostatic shaker. The adsorption temperature was adjusted with the thermostatic shaker and set at a fixed speed of 170 rpm/min. After adsorption, the sample (3 mL) was filtered through a 0.45 μm filter membrane, and the *p*-nitrophenol concentration was measured at 310 nm using a UV-1000 UV-Vis spectrophotometer (Shunyu Hengping Scientific Instruments Co., Ltd., Shanghai, China).

The adsorption capacity was measured using Equation (10):

(10) $$q_{t}=\frac{\left(C_{0}-C_{t}\right)\cdot V}{m}$$

where *q*_*t*_—the adsorption capacity of the *p*-nitrophenol at time (t), (mg/g); *C*_*t*_—the remaining concentration in the *p*-nitrophenol solution at time (t), (mg/L); *C*_0_—the initial concentration of the *p*-nitrophenol, (mg/L); *V*—the volume of the *p*-nitrophenol solution (L); *m*—the mass of the adsorbent (g).

### 3.5. Regeneration Method

After the batch adsorption experiments, the suspension was centrifuged to extract the saturated adsorbent. The obtained adsorbent was swept for 4 h at 200 °C with 4 L/min of hot air. The reusability of the adsorbent was studied for five cycles at a temperature of 298 K and initial concentration of 50 mg/L.

## 4. Conclusions

This study successfully prepared ZIF-8-PhIm via the SALE method for *p*-nitrophenol removal from water. At a temperature of 298 K and initial concentration of 400 mg/L, ZIF-8-PhIm demonstrated a higher adsorption capacity (828.29 mg/g) than ZIF-8 due to the increased pore size, capacity and hydrophilicity, which exposed more active sites for adsorption. The mechanism of *p*-nitrophenol adsorption mainly involved hydrogen bonding, π-π interaction and weaker electrostatic interaction. The Langmuir isothermal and pseudo-second-order kinetic models were used to characterize the *p*-nitrophenol adsorption by ZIF-8-PhIm, with an activation energy (Ea) of 11.54 kJ/mol. The adsorption process was found to be single molecular layer physical adsorption. After five regeneration cycles at a temperature of 298 K and an initial concentration of 50 mg/L, the adsorption capacity of ZIF-8-PhIm stabilised at 105.43 mg/g, indicating good reusability.

## Figures and Tables

**Table 1:** Parameters of the pore structure.

Sample	Surface Area (m^2^/g)	Average Pore Diameter (nm)	Pore Volume (cm^3^/g)	Micropore Volume (cm^3^/g)	Mesopore Volume (cm^3^/g)	Mesopore Volume Percentage (%)
ZIF-8-PhIm	1663.79	2.08	0.85	0.54	0.31	36.47
ZIF-8	1700.39	1.85	0.79	0.60	0.19	24.05

**Table 3:** Parameters of the isotherm models for *p*-nitrophenol adsorption on ZIF-8-PhIm.

Model		298 K	308 K	318 K
Langmuir	q_m_ (mg/g)	1105.04	1108.91	1117.72
	K_L_ (L/mg)	0.03	0.03	0.03
	R^2^	0.981	0.985	0.987
Freundlich	1/n	0.53	0.52	0.50
	K_F_ ((mg/g)(mg/L)^1/n^)	69.54	77.37	87.26
	R^2^	0.926	0.928	0.925

**Table 4:** Thermodynamic parameters of *p*-nitrophenol adsorption by ZIF-8-PhIm.

T (K)	ΔG (kJ/mol)	ΔH (kJ/mol)	ΔS (J/(K∙mol))
298.15	−1.98	27.85	95.45
318.15	−3.89		

**Table 5:** Parameters of the kinetic models for the adsorption of ZIF-8-PhIm.

Model		298 K	308 K	318 K
Pseudo-first-order kinetic model	k_1_ (min^−1^)	0.23	0.25	0.29
	q_e1, cal_ (mg/g)	125.12	126.61	127.28
	q_e1, exp_ (mg/g)	129.51	131.27	128.27
	R^2^	0.969	0.971	0.973
Pseudo-second-order kinetic model	k_2_ (g/(mg^.^min))	2.4 × 10^−3^	2.7 × 10^−3^	3.3 × 10^−3^
	q_e2, cal_ (mg/g)	133.41	134.32	134.35
	q_e2, exp_ (mg/g)	129.51	131.27	132.70
	R^2^	0.989	0.992	0.991

**Table 6:** Binding energies and relative contents in the O 1s profiles of ZIF-8-PhIm before and after *p*-nitrophenol adsorption.

Species		Before Adsorption		After Adsorption	
		Binding Energy (eV)	Atom (%)	Binding Energy (eV)	Atom (%)
O 1s	Zn-O	530.80		530.80	65.83
	Zn-OH	531.71	87.08	531.71	22.94
	H_2_O	532.98	12.92	532.99	11.23

## Data Availability

The data are contained within the article or the Supplementary Materials.

## Supplementary Materials

The following supporting information can be downloaded at: https://www.mdpi.com/article/10.3390/molecules28104195/s1, Figure S1: Adsorption capacity of zif-8 synthesized by different zinc nitrate:2-methylimidazole ratios. Figures S2: Adsorption capacity of ZIF-8-PhIm synthesized with different ZIF-8:2-phenylimidazole molar ratios. Figure S3: ^1^H NMR spectra (in Trifluoroacetic Acid-d) of ZIF-8 and ZIF-8-PhIm. Figure S4: Contact angles of ZIF-8-PhIm and ZIF-8. Figure S5: TGA and DTA curves of ZIF-8-PhIm. Figure S6: Activation energy fitting straight line for adsorption of ZIF-8-PhIm.

## Sample Availability

Samples of the compounds are not available from the authors.

## References

[1] Zhu S.Y., Niu W.X., Li H.J., Han S., Xu G.B. (2009). Single-walled carbon nanohorn as new solid-phase extraction adsorbent for determination of 4-nitrophenol in water sample. Talanta.

[2] Busca G., Berardinelli S., Resini C., Arrighi L. (2008). Technologies for the removal of phenol from fluid streams: A short review of recent developments. J. Hazard. Mater..

[3] Tchieno F.M.M., Tonle I.K. (2018). P-Nitrophenol determination and remediation: An overview. Rev. Anal. Chem..

[4] Lazo-Cannata J.C., Nieto-Márquez A., Jacoby A., Paredes-Doig A.L., Romero A., Sun-Kou M.R., Valverde J.L. (2011). Adsorption of phenol and nitrophenols by carbon nanospheres: Effect of pH and ionic strength. Sep. Purif. Technol..

[5] Extremera R., Pavlovic I., Pérez M.R., Barriga C. (2012). Removal of acid orange 10 by calcined Mg/Al layered double hydroxides from water and recovery of the adsorbed dye. Chem. Eng. J..

[6] Sun Y.Y., Zhou J.B., Cai W.Q., Zhao R.S., Yuan J.P. (2015). Hierarchically porous NiAl-LDH nanoparticles as highly efficient adsorbent for *p*-nitrophenol from water. Appl. Surf. Sci..

[7] Ania C.O., Cabal B., Pevida C., Arenillas A., Parra J.B., Rubiera F. (2007). Removal of naphthalene from aqueous solution on chemically modified activated carbons. Water Res..

[8] Kumar N., Reddy L., Parashar V., Ngila J.C. (2017). Controlled synthesis of microsheets of ZnAl layered double hydroxides hexagonal nanoplates for efficient removal of Cr(VI) ions and anionic dye from water. J. Environ. Chem. Eng..

[9] Ahmadi S.A.R., Kalaee M.R., Moradi O., Nosratinia F., Abdouss M. (2022). Synthesis of novel zeolitic imidazolate framework (ZIF-67)-zinc oxide (ZnO) nanocomposite (ZnO@ ZIF-67) and potential adsorption of pharmaceutical (tetracycline (TCC)) from water. J. Mol. Struct..

[10] Zhao Y.B., Wang L., Zhu L.C., Gao F., Xu X.R., Yang J.Y. (2022). Removal of *p*-nitrophenol from simulated sewage using steel slag: Capability and mechanism. Environ. Res..

[11] Santos A.D., Viante M.F., Pochapski D.J., Downs A.J., Almeida C.A.P. (2018). Enhanced removal of *p*-nitrophenol from aqueous media by montmorillonite clay modified with a cationic surfactant. J. Hazard. Mater..

[12] Álvarez-Torrellas S., Martin-Martinez M., Gomes H.T., Ovejero G., García J. (2017). Enhancement of *p*-nitrophenol adsorption capacity through N_2_-thermal-based treatment of activated carbons. Appl. Surf. Sci..

[13] Lgaz H., Lee H.S. (2021). Computational investigation on interaction mechanism of sulfur mustard adsorption by zeolitic imidazolate frameworks ZIF-8 and ZIF-67: Insights from periodic and cluster DFT calculations. J. Mol. Liq..

[14] Petit C., Bandosz T.J. (2012). Exploring the coordination chemistry of MOF-graphite oxide composites and their applications as adsorbents. Dalton Trans..

[15] Sun X.J., Xia Q.B., Zhao Z.X., Li Y.W., Li Z. (2014). Synthesis and adsorption performance of MIL-101(Cr)/graphite oxide composites with high capacities of n-hexane. Chem. Eng. J..

[16] Lin K.Y.A., Hsieh Y.T. (2015). Copper-based metal organic framework (MOF), HKUST-1, as an efficient adsorbent to remove *p*-nitrophenol from water. J. Taiwan Inst. Chem. Eng..

[17] Miao H.X., Song S.Y., Chen H., Zhang W.H., Han R.P., Yang G. (2020). Adsorption study of *p*-nitrophenol on a silver (I) triazolate MOF. J. Porous Mater..

[18] Jia Z.Q., Jiang M.C., Wu G.R. (2017). Amino-MIL-53(Al) sandwich-structure membranes for adsorption of *p*-nitrophenol from aqueous solutions. Chem. Eng. J..

[19] Stephenson C.J., Hupp J.T., Farha O.K. (2016). Postassembly transformation of a catalytically active composite material, Pt@ ZIF-8, via solvent-assisted linker exchange. Inorg. Chem..

[20] Pullen S., Fei H., Orthaber A., Cohen S.M., Ott S. (2013). Enhanced photochemical hydrogen production by a molecular diiron catalyst incorporated into a metal-organic framework. J. Am. Chem. Soc..

[21] Bury W., Fairen-Jimenez D., Lalonde M.B., Snurr R.Q., Farha O.K., Hupp J.T. (2013). Control over catenation in pillared paddlewheel metal–organic framework materials via solvent-assisted linker exchange. Chem. Mater..

[22] Kenyotha K., Chanapattharapol K., Mccloskey S., Jantaharn P. (2020). Water based synthesis of ZIF-8 assisted by hydrogen bond acceptors and enhancement of CO_2_ uptake by solvent assisted ligand exchange. Crystals.

[23] Li M.H., Liu Y.B., Li F., Shen C.S., Kaneti Y.V., Yamauchi Y., Yuliarto B., Chen B., Wang C.C. (2021). Defect-Rich hierarchical porous UiO-66(Zr) for tunable phosphate removal. Environ. Sci. Technol..

[24] Chen Z.C., Zhang H.W., Fan G.Y., He X.Y., He Z.B., Zhang L.H. (2022). Diatomite composited with a zeolitic imidazolate framework for removing phosphate from water. ACS Omega.

[25] Yu D.B., Shao Q., Song Q.J., Cui J.W., Zhang Y.L., Wu B., Ge L., Wang Y., Zhang Y., Qin Y.Q. (2020). A solvent-assisted ligand exchange approach enables metal-organic frameworks with diverse and complex architectures. Nat. Commun..

[26] Tsai C.W., Niemantsverdriet J.W., Langner E.H.G. (2018). Enhanced CO_2_ adsorption in nano-ZIF-8 modified by solvent assisted ligand exchange. Microporous Mesoporous Mater..

[27] Amrhar O., Gana L.E., Mobarak M. (2021). Calculation of adsorption isotherms by statistical physics models: A review. Environ. Chem. Lett..

[28] Abbasi A.R., Moshtkob A., Shahabadi N., Masoomi M.Y., Morsali A. (2019). Synthesis of nano zinc-based metal–organic frameworks under ultrasound irradiation in comparison with solvent-assisted linker exchange: Increased storage of N_2_ and CO_2_. Ultrason. Sonochem..

[29] Shi Y.N., Zhang X.F., Liu H.T., Han J.Y., Yang Z.J., Gu L., Tang Z.Y. (2020). Metalation of catechol-functionalized defective covalent organic frameworks for lewis acid catalysis. Small.

[30] Massahud E., Ahmed H., Babarao R., Ehrnst Y., Alijani H., Darmanin C., Murdoch B.J., Rezk A., Yeo L.Y. (2023). Acoustomicrofluidic defect engineering and ligand exchange in ZIF-8 metal–organic frameworks. Small Methods.

[31] Gao S., Hou J.W., Deng Z.Y., Wang T.S., Beyer S., Buzanich A.G., Richardson J.J., Rawal A., Seidel R., Zulkifli M.Y. (2019). Improving the acidic stability of zeolitic imidazolate frameworks by biofunctional molecules. Chem.

[32] Ma H.F., Xu Z.G., Wang W.Y., Gao X., Ma H.F. (2019). Adsorption and regeneration of leaf-based biochar for *p*-nitrophenol adsorption from aqueous solution. RSC Adv..

[33] Aldawsari A.M., Alsohaimi I.H., Hassan H.M.A., Berber M.R., Abdalla Z.E.A., Hassan I., Saleh E.A.M., Hameed B.H. (2020). Activated carbon/MOFs composite: AC/NH_2_-MIL-101(Cr), synthesis and application in high performance adsorption of *p*-nitrophenol. J. Saudi Chem. Soc..

[34] Cheng M., Jiang J., Wang J.J., Fan J. (2019). Highly salt resistant polymer supported ionic liquid adsorbent for ultrahigh capacity removal of *p*-nitrophenol from water. ACS Sustainable Chem. Eng..

[35] Sun Y.Y., Zhou J.B., Cheng Y., Yu J.G., Cai W.Q. (2014). Hydrothermal synthesis of modified hydrophobic Zn-Al-layered double hydroxides using structure-directing agents and their enhanced adsorption capacity for *p*-nitrophenol. Adsorpt. Sci. Technol..

[36] Gao F., Xu X.R., Yang J.Y. (2022). Removal of *p*-nitrophenol from simulated sewage using MgCo-3D hydrotalcite nanospheres: Capability and mechanism. RSC Adv..

[37] Langmuir I. (1918). The adsorption of gases on plane surfaces of glass, mica and platinum. J. Am. Chem. Soc..

[38] Duranoğlu D., Trochimczuk A.W., Beker U. (2012). Kinetics and thermodynamics of hexavalent chromium adsorption onto activated carbon derived from acrylonitrile-divinylbenzene copolymer. Chem. Eng. J..

[39] Ho Y.S., McKay G. (1999). Pseudo-second order model for sorption processes. Process Biochem..

[40] Weber W.J., Morris J.C. (1963). Kinetics of adsorption on carbon from solution. J. Sanit. Eng. Div..

[41] Milmile S.N., Pande J.V., Karmakar S., Bansiwal A., Chakrabarti T., Biniwale R.B. (2011). Equilibrium isotherm and kinetic modeling of the adsorption of nitrates by anion exchange Indion NSSR resin. Desalination.

[42] Wang J., Xiang L. (2014). Formation of ZnO rods with varying diameters from ε-Zn (OH)_2_. J. Cryst. Growth.

[43] Ge X., Song X.Y., Ma Y., Zhou H.J., Wang G.Z., Zhang H.M., Zhang Y.X., Zhao H.J., Wong P.K. (2016). Fabrication of hierarchical iron-containing MnO_2_ hollow microspheres assembled by thickness-tunable nanosheets for efficient phosphate removal. J. Mater. Chem. A.

[44] Li J., Wu Y., Li Z., Zhang B., Zhu M., Hu X., Zhang Y.M., Li F.T. (2014). Zeolitic imidazolate framework-8 with high efficiency in trace arsenate adsorption and removal from water. J. Phys. Chem. C.

[45] Zhang Z., Chen Y., Hu C.Y., Zuo C., Wang P., Chen W.Q., Ao T.Q. (2021). Efficient removal of tetracycline by a hierarchically porous ZIF-8 metal organic framework. Environ. Res..

[46] Li C.H., Wang F.L., Xu X., Shi Y.B., Liang J.S., Yang R.S., Liu J., Zhao Z.L. (2023). A high-capacity malleable cellulose aerogel with layered double hydroxide decorating ZIF-8 for efficient adsorption of ciprofloxacin. Chem. Eng. J..

[47] Jung B.K., Hasan Z., Jhung S.H. (2013). Adsorptive removal of 2, 4- dichlorophenoxyacetic acid (2, 4-D) from water with a metal–organic framework. Chem. Eng. J..

[48] Fraga T.J.M., Ghislandi M.G., Carvalho M.N., Sobrinho M.A.D.M. (2020). One step forward: How can functionalization enhance the adsorptive properties of graphene towards metallic ions and dyes?. Environ. Res..

[49] Fraga T.J.M., Carvalho M.N., Ghislandi M.G., Sobrinho M.A.D.M. (2019). Functionalized graphene-based materials as innovative adsorbents of organic pollutants: A concise overview. Braz. J. Chem. Eng..

[50] Fraga T.J.M., Carvalho M.N., Fraga D.M.S.M., Silva M.D.C.L.D., Ferreira J.M., Sobrinho M.A.D.M. (2020). Treated residue from aluminium lamination as adsorbent of toxic reactive dyes-a kinetic, equilibrium and thermodynamic study. Environ. Technol..

