# Supplementary material for: Removal of p-Nitrophenol by Adsorption with 2-Phenylimidazole-Modified ZIF-8
Source: Molecules. 2023 May 19;28(10):4195. doi: 10.3390/molecules28104195 (PMC10224213; doi:10.3390/molecules28104195)
Supplement: Supplementary file 1 [file molecules-28-04195-s001.zip › molecules-2391541-supplementary.pdf]

Supplementary Materials

Removal of p-nitrophenol by adsorption with 2-phenylimidazole modified ZIF-8

Yu Zhao[1], Peiqing Yuan[2], Xinru Xu[1], Jingyi Yang[1,*]

[1] *International Joint Research Center of Green Energy Chemical Engineering, East China University of Science and Technology, Meilong Road 130, Shanghai, PR China, 200237*

[2] *State Key Laboratory of Chemical Engineering, East China University of Science and Technology, Meilong Road 130, Shanghai, PR China, 200237*

* Corresponding author. Email: jyyang@ecust.edu.cn (Jingyi Yang)

Text S1 Optimization of synthesis

The synthesis process of the samples was optimized. Firstly, the synthesis of ZIF-8 was optimized and the ratios of 1:2, 1:4 and 1:8 of zinc nitrate to 2-methylimidazole were selected for p-nitrophenol adsorption experiments. The synthetic yield of ZIF-8 in the ratio of 1:2 was low, so the synthetic ZIF-8 in the ratio of 1:4 and 1:8 was chosen for the comparison of adsorption experiments. The results showed that the ZIF-8 synthesized in 1:4 ratio had better adsorption properties. So the 1:4 ratio synthesized ZIF-8 was used for the subsequent modification.

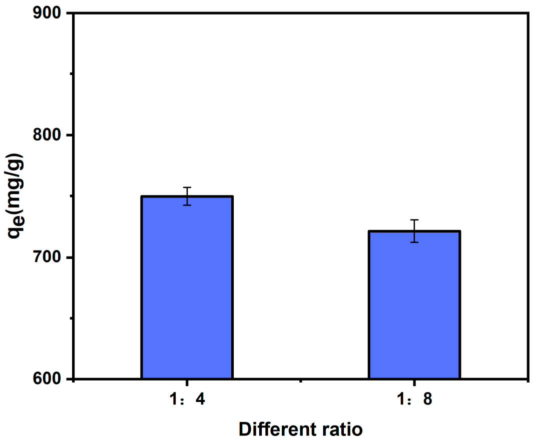

Figure S1. Adsorption capacity of zif-8 synthesized by different zinc nitrate:2-methylimidazole ratios

Then the molar ratios of ZIF-8 and 2-phenylimidazole were optimized. The results showed that the adsorption of ZIF-8-PhIm synthesized in the ratio of 1:5 was the best, so the adsorption experiments were carried out using ZIF-8-PhIm synthesized in this molar ratio.

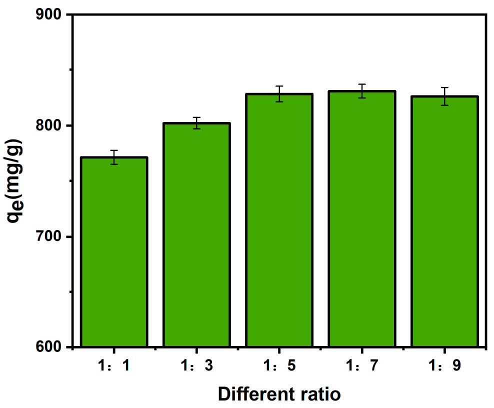

Figure S2. Adsorption capacity of ZIF-8-PhIm synthesized with different ZIF-8:2-phenylimidazole molar ratios

Text S2

^{1}H NMR spectra, Contact angles and TGA and DTA curves

For ZIF-8-PhIm, the signal of the CH=CH proton in 2-methylimidazole was observed at 7.19 ppm. The key signals for the neighbor substitution proton (2',6') and the imidazole aromatic proton (4,5) of 2 phenylimidazole were observed at 7.73 and 7.38 ppm, confirming the substitution of 2-methylimidazole by 2-phenylimidazole during SALE. The exchange ratio is approximately 1:0.04 based on the integral ratio of the CH=CH proton of 2-methylimidazole (7.19 ppm, 2H) to the ortho substituted proton of 2 phenylimidazole (7.73 ppm, 2H).

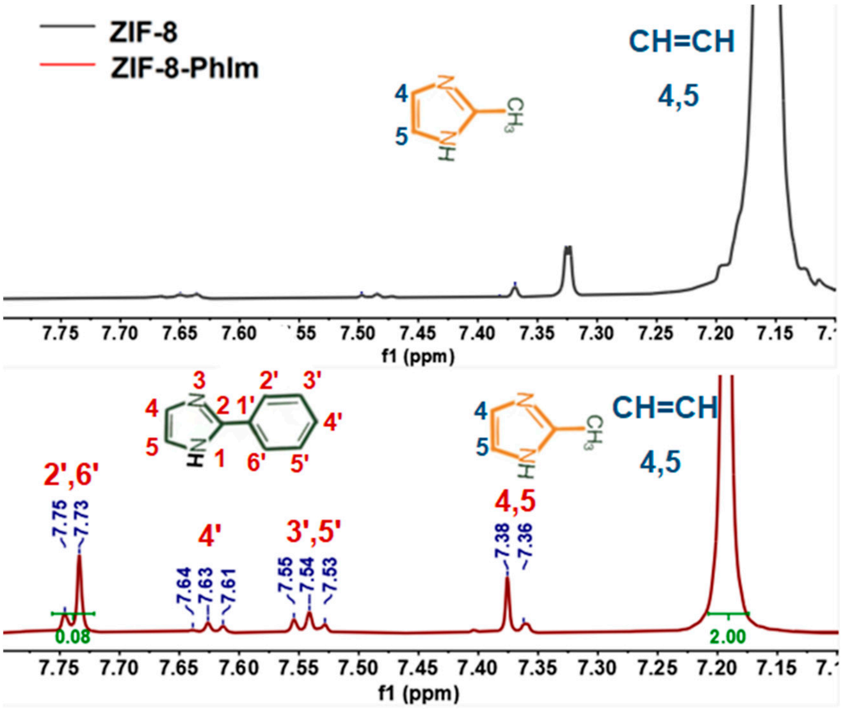

Figure S3. ^{1}H NMR spectra (in Trifluoroacetic Acid-d) of ZIF-8 and ZIF-8-PhIm.

The droplet image (Fig. S2) shows that, unlike ZIF-8 (84.66°), the contact angle of ZIF-8-PhIm is reduced by about 10° to 74.42°.

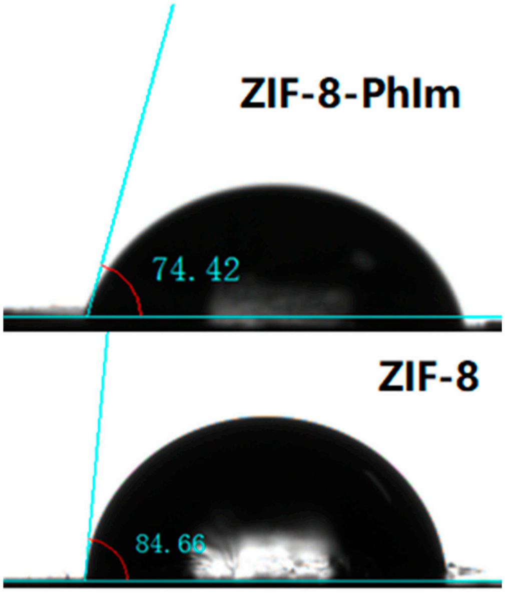

Figure S4. Contact angles of ZIF-8-PhIm and ZIF-8.

From the TGA curve, it can be seen that ZIF-8-PhIm has certain thermal stability. Before 500 °C, ZIF-8-PhIm has only 2.7 % weight loss, which is mainly due to the loss of free/crystalline water and guest molecules that are not involved in coordination.

After this, ZIF-8-PhIm started to decompose while the rate of weight loss gradually accelerated. From the DTA curves, it can be seen that the weight loss rate was faster when the temperature was increased to 601.1 °C and 737 °C. The carbon and zinc elements in its organometallic framework react with oxygen to form carbon dioxide and zinc oxide, respectively.

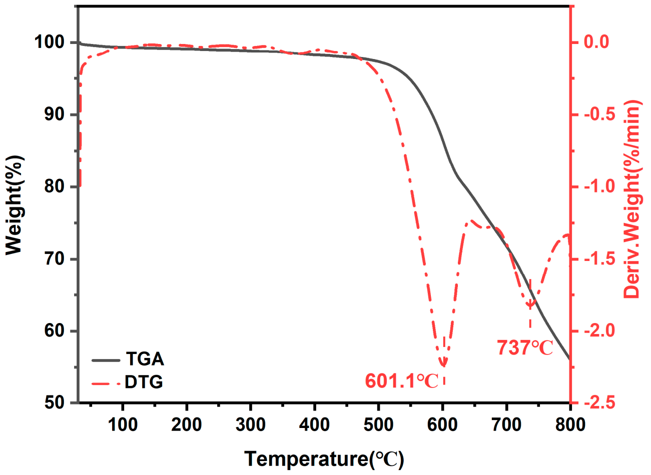

Figure S5. TGA and DTA curves of ZIF-8-PhIm.

Text S3

Calculation of activation energy for adsorption

The activation energy Ea, obtained by fitting, is 11.54 kJ/mol.

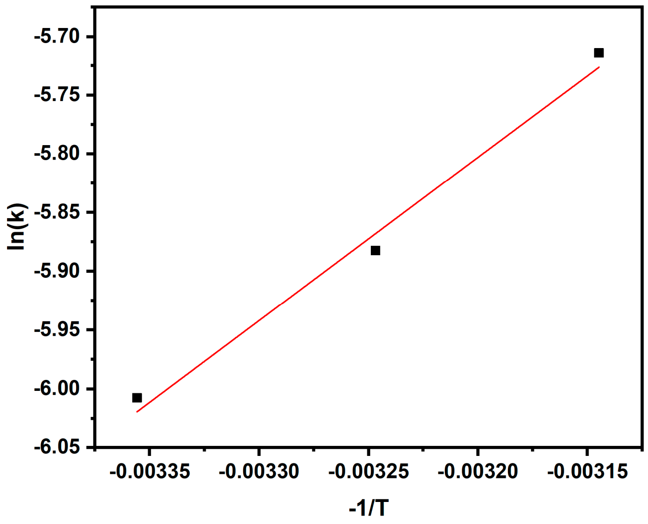

Figure S6. Activation energy fitting straight line for adsorption of ZIF-8-PhIm
